# Descriptions of Mature Larvae of the Bee Tribe Emphorini and Its Subtribes (Hymenoptera, Apidae, Apinae)

**DOI:** 10.3897/zookeys.148.1839

**Published:** 2011-11-21

**Authors:** Jerome G. Rozen, Jr.

**Affiliations:** 1 Division of Invertebrate Zoology, American Museum of Natural History, Central Park West at 79th St., New York, NY 10024, USA

**Keywords:** Emphorini, Emphorina, Ancyloscelidina, larva, last larval instar

## Abstract

A description of the mature larvae of the bee tribe Emphorini based on representatives of six genera is presented herein. The two included subtribes, Ancyloscelidina and Emphorina, are also characterized and distinguished from one another primarily by their mandibular anatomy. The anatomy of abdominal segments 9 and 10 is investigated and appears to have distinctive features that distinguish the larvae of the tribe from those of related apine tribes.

## Introduction

A recent study of the immature stages of the Exomalopsini (Rozen in press) presented a preliminary tribal key based on last larval instars to the non-cleptoparasitic apine taxa whose larvae were available, exclusive of the corbiculate tribes. It revealed that the last stage larva of *Ancyloscelis apiformis* (Fabricius) was in certain ways remarkably different from those of other Emphorini. To investigate these differences the present paper describes the tribe based on its mature larvae and then offers a larval description of *Ancyloscelis* (based primarily on *Ancyloscelis apiformis*), the only genus in the subtribe Ancyloscelidina, and compares it with a characterization of mature larvae of the subtribe Emphorina as listed in Table 1. Although Roig-Alsina and Michener (1993) first proposed the subtribe Ancyloscelina for *Ancyloscelis*, the tribal name was corrected as Ancyloscelidina and validated by Engel and Michener in Engel (2005). These are the only two subtribes of the Emphorini (Michener 2007).

**Table 1.** Taxa of the Emphorini whose mature larvae were examined for current study, with source of material and other information

EMPHORINI	
Ancyloscelidina:	
*Ancyloscelis apiformis* (Fabricius)	KU and AMNH collections
Emphorina:	Michener, 1953; AMNH collection
*Diadasia (Diadasia) enavata* (Cresson)	AMNH collection
*Diadasia (Dasiapis) olivacea* (Cresson)	“
*Diadasia (Coquillettapis) rinconis* Cockerell	“
*Diadasia (Coquillettapis) vallicola* Timberlake	“
*Diadasina (Diadasina)* sp.	“
*Melitoma grisella* (Cockerell & Porter)	“
*Melitoma marginella* (Cresson)	“
*Melitoma segmentaria* (Fabricius)	“
*Ptilothrix bombiformis* (Cresson)	Michener, 1953; AMNH collection
*Ptilothrix near sumichrasti* (Cresson)	AMNH collection
*Ptilothrix tricolor* (Friese)	“
*Toromelissa nemaglossa* (Toro & Ruz)	“

With great pleasure I dedicate this study to Drs. Kumar and Valerie Krishna, long-term associates and currently next-door office neighbors, whom I have known for nearly a half century. May their good humor and scholarship prevail long into the future!

Aspects of the biology of *Ancyloscelis apiformis* were described by Torchio (1974), Michener (1974), and Rozen (1984), and more recently Gonzalez et al. (2007) treated the biology of *Ancyloscelis aff. apiformis*. Previous descriptions of immature stages were listed by McGinley (1989).

In the study of larval Exomalopsini, the highly sclerotized mandibular morphology revealed considerable structural variation; this variation was not reflected in the surrounding mouthparts, presumably because of their soft, non-sclerotized anatomy. A preliminary survey of emphorine larval mandibles from the earlier study revealed mandibular variation as remarkable as that of the Exomalopsini, thus prompting the current study.

## Methods and terminology

**Figures 1–3.**
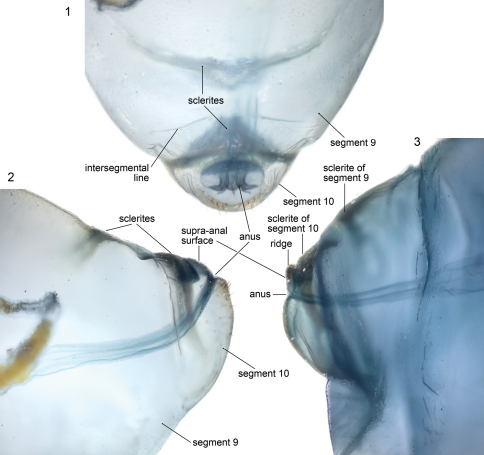
Microphotographs of terminal abdominal segments of cleared, stained emphorine larvae. **1, 2** *Melitoma grisella*, predefecating, dorsal and lateral views **3** *Diadasia rinconis*, postdefecating, lateral view.

For clearing, larvae were boiled in an aqueous solution of sodium hydroxide, stained with Chlorazol Black E, and then submerged in glycerin on well slides for study and storage. Specimens to be examined with a Hitachi S-4700 scanning electron microscope (SEM) were critical-point dried and then coated with gold/palladium. Microphotographs of Figs 1–3 were taken with a Microptics-USA photographic system equipped with an Infinity Photo-Optic K-2 lens system. Microphotographs of mandibles were taken with a Cannon PowerShot SD880 IS handheld to the ocular of a Zeiss compound microscope. Fig. 12 was rendered with a Carl Zeiss LSM 710 confocal microscope.

**Figures 4–8.**
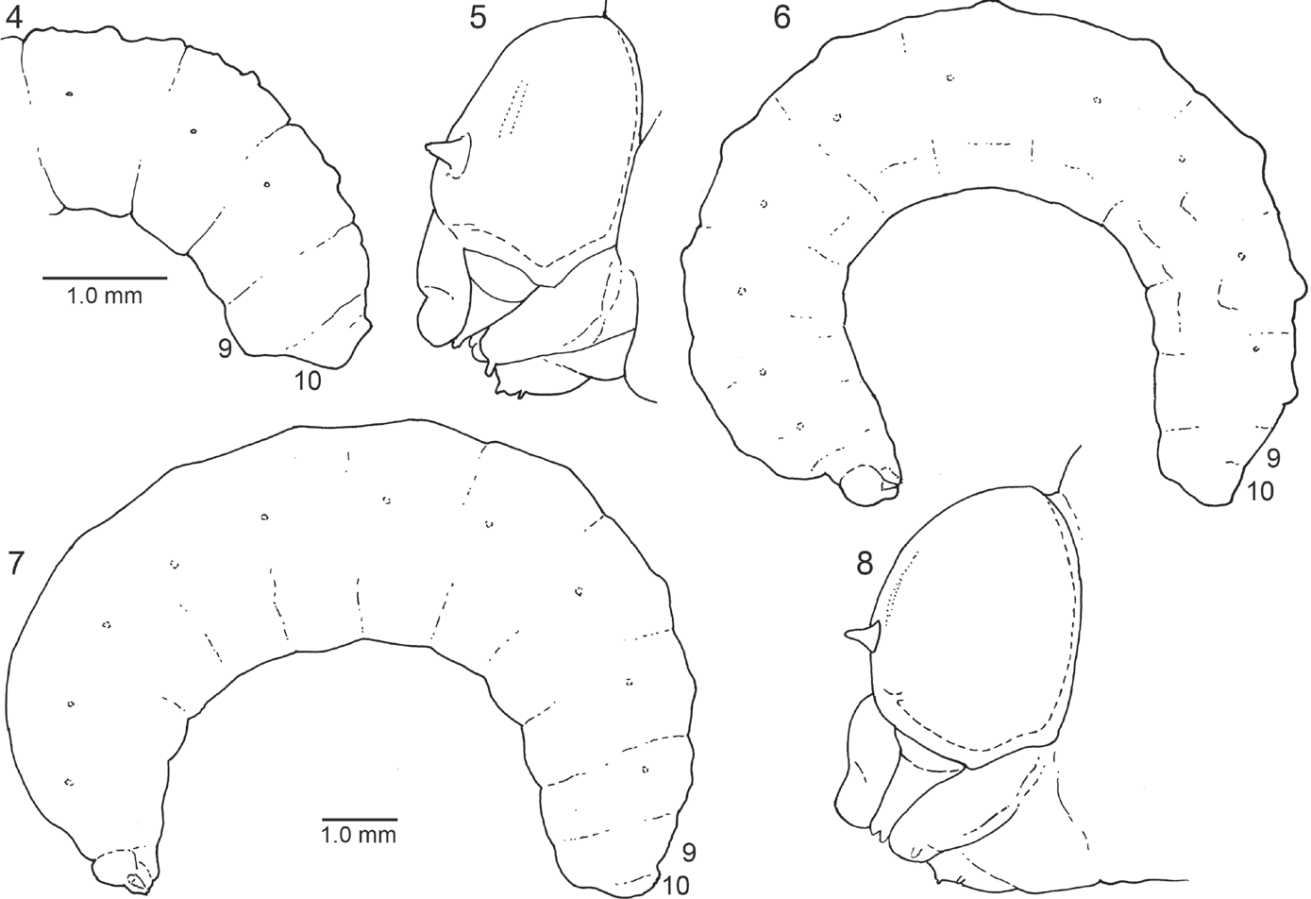
4–5. Diagrams of mature larva of *Ancyloscelis apiformis*, lateral view**4** Posterior part of abdomen of predefecating form **5** Head. **6–8.** Diagrams of last larval instar of *Diadasia rinconis*, lateral view **6** Predefecating form **7** Postdefecating form **8**Head, lateral view; Figs 6 and 7 to same scale.

**Figures 9–12.**
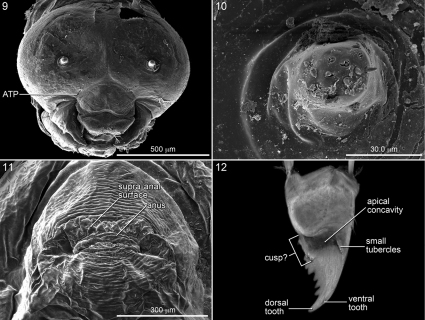
9–11. SEM micrographs of last larval instar of *Ancyloscelis apiformis.* **9** Head, frontal view **10** Antenna with many sensilla **11** Abdominal segment 10, posterior view. **12.** Confocal micrograph of mandible of same, ventral view.

Table 1 gives the full name and authorship of all species treated herein.

For descriptions of mandibles, the right mandible is used and assumed to have its long axis horizontal making the upper surface dorsal, lower surface ventral, and inner surface the adoral surface. As explained in Rozen (in press), the cusp is defined as an adoral extension of the apical mandibular edge that forms the upper boundary of the apical concavity. It seems well represented in the Emphorina but greatly modified in Ancyloscelidina because of the blade-like thinness of the mandibular apex and the coarse serrations of the dorsal apical edge (Figs 12, 21, 22). The ventrally projecting tubercle-like structure and surrounding uneven surface (Figs 12, 21) near the basal boundary of the apical concavity is likely a derived modification of the cusp.

To determine the foramen-to-head-width index of mature larvae, the maximum transverse width of the foramen was divided by the maximum transverse head width. This is a measure of the degree of constriction of the posterior edge of the head capsule relative to the lateral expansion of the parietals.

## Anatomy of Abdominal Apex

This section explores the anatomy of abdominal segments 9 and 10 of the emphorine last larval instar because certain features found there have been overlooked. Although this study is based primarily on the predefecating larva of *Melitoma grisella* (Cockerell and Porter) and *Diadasia rinconis* Cockerell, these features are evident on all emphorine larvae examined. Abdominal segments 9 and 10 each has a scarcely visible dorsal sclerotized area that is nearly unpigmented. However, when a specimen is cleared and stained with Chlorazol Black E, the sclerite of abdominal segment 9 is visible (although poorly delineated) as a narrow, transverse, somewhat impressed (compare with surrounding integument) dark band (Figs 1, 2) tapering at both ends and stretching across the segment somewhat more than halfway to the segment’s posterior edge. Although its anterior margin in gently curved, its posterior margin is broadly V-shaped and points toward the following segment.

The stained transverse sclerite (also not sharply delineated) of abdominal segment 10 rings much of the segment but fades ventrally. Its anterior margin approaches the preceding segment at mid line, so that the sclerites of abdominal segments 9 and 10 point toward one another suggesting that they function together. The dorsal part of the posterior edge of the sclerite on segment 10 bends outward forming a shallow groove in front of it. The abdominal apex lies beyond this sclerite, and the anus (Figs 1, 11, 16, 17) is a transverse slit, positioned a short distance posterior to the sclerite. The surface of the abdominal apex between anus and sclerite projects beyond the sclerite as the raised, verrucose supra-anal surface (Figs 11, 16, 17) with its dorsal edge forming a semicircle from one side of the anus to the other when viewed from behind (Figs 11, 16, 17). This edge often becomes carinate on postdefecating specimens creating a ridge circling the anus from above (Figs 11, 16). The area below the anus is planar, defined as a semicircle by the conspicuous setae at the border in the case of *Melitoma grisella* (Figs 1, 2, 17); in other species that area is less well-defined. Hence, the dorsal view of the abdominal apex is an oval, traversed by the anus (Fig. 1).

The dorsal sclerites of abdominal segments 9 and 10 and the position of the anus with projecting, verrucose surface, all ringed by fine setae, suggest that these structures function together for some purpose currently not understood. One can speculate that these modifications support fecal deposition or perhaps deposition of some substance on the cell wall to safeguard the bee from water loss or parasites. Instead, these features might relate in some way to locomotion, for how does such an elongate larva move around in the cell while it feeds and defecates? Careful observations of living specimens during this stadium will likely lead to an explanation.

## Mature larva of Emphorini

**Diagnosis:** The best way to distinguish larval emphorines from those of other apid tribes is with the characters indicated in the preliminary tribal key based on last larval instars to the non-cleptoparasitic apine taxa (Rozen in press). The presence of fine to moderate setae on abdominal segment 10 is a feature restricted to the Emphorini and to the exomalopsine genus *Eremapis* among the Apidae, but *Eremapis* lacks the sclerites of abdominal segments 9 and 10. Unlike other emphorines, *Toromelissa nemaglossa* (Toro and Ruz), known from Chile, has only a pair of setae on the outer surface of its mandible, although like other emphorine taxa it does possess numerous scattered fine setiform sensilla on abdominal segment 10 as well as a spiculate mandibular corium. No other bee larva is known to possess this combination of characters. The following is based on mature larvae of species listed in Table 1, which also indicates the sources of preserved specimens.

**Figures 13–16.**
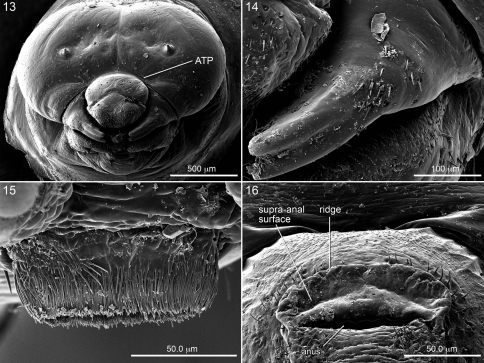
SEM micrographs of postdefecating larva of *Diadasia rinconis*. **13** Head, frontal view **14** Left mandible, showing setae on outer surface **15** Salivary lips, from above **16** Upper part of segment 10, posterior view.

*Head* (Figs 5, 8, 9, 13): Integument of head capsule with scattered, small sensilla, many of which are clearly setiform; epipharyngeal surface spiculate but with different patterns of distribution; mandibular corium nonspiculate, except clearly spiculate in *Toromelissa* and in some *Diadasia*. Integument pigmentation variable; mandible pigmented apically but far less so basally, with pigmented area usually defined by sharp line of separation (Figs 24, 26, 28, 30, 32, 34); hypopharyngeal groove distinct.

Head (Figs 6, 7) moderately to very small relative to elongate body (Figs 6, 7); width of foramen magnum compared to head width as follows: *Ancyloscelis* 0.73; *Diadasia* 0.66–0.70; *Diadasina* 0.67; *Melitoma* 0.65–0.72; *Ptilothrix* 0.71; *Toromelissa* 0.71; bridge between posterior tentorial pits well developed; rest of tentorium normally robust for cocoon-spinning larva (even though not all spin cocoons). Center of anterior tentorial pit much closer to anterior mandibular articulation than to outer ring of antenna in frontal view (Figs 9, 13; ATP = anterior tentorial pit), so that lateral segment (between anterior tentorial pit and anterior mandibular articulation) of epistomal ridge extremely short; posterior tentorial pit (i.e., junction point of postoccipital ridge, hypostomal ridge, and tentorial bridge) in normal position, deeply recessed; all internal head ridges strongly developed; coronal ridge extending to, or nearly to, middle of epistomal ridge in frontal view; median section of epistomal ridge more or less well developed; dorsomedial portion of postoccipital ridge nearly straight (not bending forward) as viewed from above; hypostomal ridge without distinct dorsal ramus. Parietal bands faintly evident as integumental scars. Antennal prominence non-extant;antennal papilla moderate to large in size, always longer than basal diameter, conical in shape, apically bearing 6 or more (in some cases many more) sensilla. Apex of labrum at most shallowly emarginated in frontal view (Figs 9, 13); apical front surface of labrum with pair of low, forward-projecting, sensilla-bearing lobes (Figs 5, 8, 9, 13); transverse labral sclerite absent.

Mandible with two apical teeth but on postdefecating forms mandible sometimes appearing to have single tooth because of wear; outer surface of mandible with 8 or more small to large setae at mid length, except *Toromelissa* with only a pair of setae; other mandibular features varying considerably between subtribes: see subtribal descriptions, below. Labiomaxillary region moderately weakly projecting in lateral view (Figs 5, 8) for cocoon spinning larva. Maxilla with apex bent adorally, bearing palpus subapically; galea not evident; cardo and stipes sclerotized but in some cases unpigmented; articulating arm of stipital sclerite evident; maxillary palpus well developed, about twice as long as labial palpus but shorter and more slender than antennal papilla. Labium clearly divided into prementum and postmentum; prementum moderately small in frontal view; premental sclerite weakly evident; labial palpus about as long as basal diameter. Salivary opening on apex of prementum, transverse with strongly (*Diadasina*, *Melitoma*, *Ptilothrix*) to weakly (*Ancyloscelis*, *Toromelissa*) projecting lips that vary in width; lips consisting of tapering elongate filaments (Fig. 15). Except in *Ancyloscelis*,hypopharynx abruptly elevated behind articulating arms of stipes, high, sometimes densely covered with coarse spicules, other times with fewer, finer spicules; hypopharyngeal groove present.

*Body:* Integument without general body setae, but abdominal segment 10 with fine to moderately conspicuous setae found especially around anus (a few setae may also be found dorsally on posterior part of segment 9); ventral surfaces of all segments with most species spiculate except for segment 10. Body form of predefecating larva (Fig. 6) unusually elongate, linear, parallel-sided; extent of expression of inter- and intrasegmental lines variable on predefecating larva (partly determined by amount of food ingested), on postdefecating larva often evident; dorsal body tubercles usually absent but see Remarks, below; dorsal tubercles absent on abdominal segment 9; abdominal segment 9 on pre- and postdefecating forms produced ventrally as seen in lateral view (Figs 4, 6, 7); abdominal segment 10 positioned dorsally on 9 in lateral view (Figs 4, 6, 7); anus positioned close to dorsal surface on segment 10 (Figs 2, 3); on postdefecating larvae, dorsal surface of segment 10 traversed by groove extending from one side of anus to other side, its posterior edge ending as strong transverse ridge above anus. Spiracles (Figs 4, 6, 7) small to moderate sized, usually inconspicuous, subequal in size throughout, not surrounded by well defined sclerites, and not on tubercles; peritreme present; atrium projecting beyond body wall, with distinct rim, globose; atrial wall smooth, without ridges or spines, moderately thick; primary tracheal opening with collar; subatrium consisting of about 12 chambers; subatrial chambers decreasing in outside diameter from body surface inward. Males to extent known (but unknown in case of *Ancyloscelis apiformis*) with single median scar on apex of ventral protuberance of abdominal segment 9; females presumably lacking scars.

**Remarks:** Although dorsal body tubercles are generally absent on mature larvae, earlier instars and even on early stages of last larval instars have paired tubercles on most body segments rising from the middle of each segment for abdominal segments 9 and 10. (These tubercles should not to be confused with the middorsal tubercles of immature Megachilidae, which are intersegmental in position, Rozen and Hall (2011) figs 85, 86.)

Each tubercle is small but often rises sharply with its front-to-back measurement about the same as the lateral measurement (i.e., tubercle non-transverse). Tubercles are uniquely positioned for bee larvae: those of each segment tend to be contiguous, lying close to the body midline. On some species they appear to be a single median bimodal tubercle.

**Figure 17–20.**
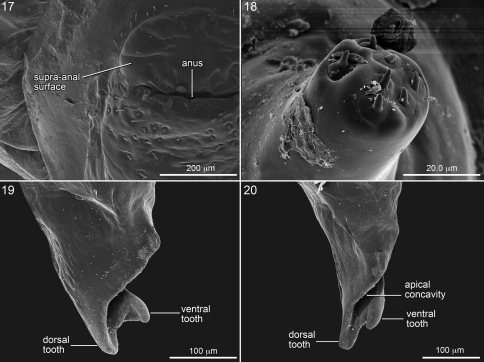
SEM micrographs of mature larva of *Melitoma grisella*. **17** Left side of abdominal segment 10, posterior view **18** Antenna **19** Mandible, dorsal view, and **20** inner view.

**Figures 21–34.**
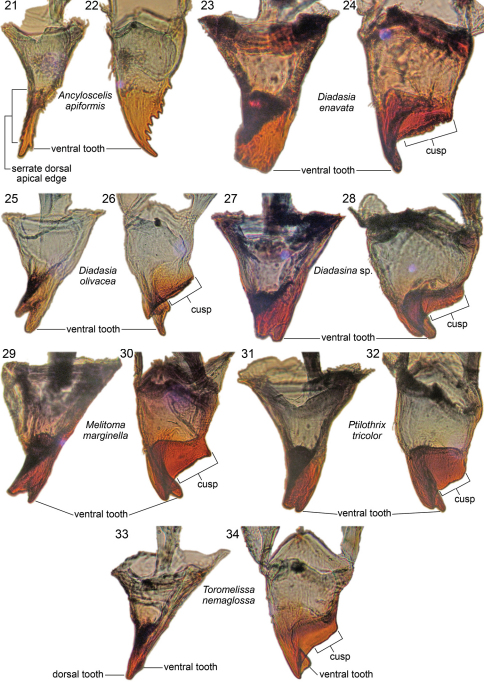
Right mandibles of mature larvae of Emphorini, showing inner view and dorsal view of each representative, as labeled.

## Mature Larva of Subtribe Ancyloscelidina

Description:*Head* (Figs 5, 9): Epipharyngeal surface with patch of short but abundant spicules covering most of anterior surface on each side; mandibular corium nonspiculate. Integument unpigmented except for mandibular apices and mandibular points of articulation with head capsule; hypopharyngeal groove faintly pigmented.

Mandibular apex uniformly pale tan, with sharp line demarking tan apex from nearly pigmentless basal part of mandible as seen in dorsal view (Fig. 22, though value contrast generally not as great as in mandible of Emphorina). Entire mandibular apex rotated and flattened, blade-like, so that coarsely serrated dorsal edge directed adorally, forming very broad, ventrally directed apical concavity (Fig. 12); dorsal apical tooth elongate, gradually narrowing to acute point directed adorally (mandible appearing rapacious) (Figs 12, 22); ventral apical tooth greatly reduced, scarcely noticeable (Figs 12, 21, 22); ventral edge of apical concavity sharply defined by fine ridge, which toward base bears short series of small tubercles (Figs 12, 21); dorsal apical edge of concavity broadening slightly toward base and bearing large, ventrally projecting tubercle and uneven surface at its base (Fig. 12); these elements presumably homologue of mandibular cusp. Cardo and stipes sclerotized but unpigmented. Prementum moderately small in frontal view. Salivary lips weakly projecting, only about one-half as wide as distance between bases of labial palpi. Hypopharynx well behind apices of articulating arms of stipes, low, questionably bilobed, faintly spiculate on both sides.

*Material examined:* 3 postdefecating larva: Trinidad: Maracas Valley, II-24-1966, III-01-1966 (F.D. Bennett, J.G. Rozen); 1 predefecating larva: same except III-08-1968 (J.G. and B.L. Rozen); 4 predefecating, 1 postdefecating larvae: Colombia: Valle del Cauca: Cali I-10-1972 (C.D. Michener).

### Mature Larva of Subtribe Emphorina

**Description:** *Head* (Figs 8, 13): Apicolateral angles of epipharyngeal surface angles with restricted swollen protuberances well separated from one another, each of which is densely covered with short spicules; mandibular corium nonspiculate, except clearly spiculate in *Toromelissa nemaglossa* and in some *Diadasia*. Integumental sclerotized areas, especially internal head ridges and sclerotized mouthparts, tending to be more pigmented than those of Ancyloscelidina.

Apical part of mandible (including mandibular apex and all of cuspal area) very darkly pigmented, almost black; line separating pigmented and nonpigmented parts sharply defined as seen in dorsal (Figs 24, 26, 28, 30, 32, 34) or ventral view. Mandibular apex usually with two apical teeth; dorsal tooth larger, ventral tooth slightly smaller (except approximately equal in *Diadasina*, Fig. 27,and in some species such as *Diadasia olivacea*, Fig. 25, ventral tooth longer than dorsal one); dorsal apical mandibular edge without teeth; ventral mandibular tooth and ventral edge of mandibular apex twisted adorally forming elongate oblique apical concavity (Fig. 20) on adoral apical surface in conjunction with strongly produced dorsal apical edge (Fig. 20); when viewed dorsally (Figs 19, 34) ventral tooth appearing more curved than dorsal tooth; adoral surface of cusp thick toward mandibular base; leading cuspal edge linear, rounded (*Ptilothrix*),or narrowly planar (*Melitoma*, *Diadasia*), without distinct spines, sometimes irregularly roughened or minutely pebbled (e.g., *Diadasia enavata*, Fig. 24). Prementum moderately small to moderately wide in frontal view. Salivary lips weakly to strongly projecting; width one-half as wide, to as wide, as distance between bases of labial palpi. Hypopharynx well behind apices of articulating arms of stipes, often dorsally projecting, in some cases bilobed, spiculate on dorsal surface.

*Material examined:* *Diadasia enavata*: 10+ larvae, all stages: USA: Washintdon: Yakima Co.: S of Granger, IX-5-1993 (E. Miliczky). *Diadasia olivacea*: 3 predefecating last larval instars: USA: Arizona: Cochise Co.: Southwestern Research Station, 5 mi S of Portal, IX-7-1773 (J.G. Rozen, M. Favreau). *Diadasia rinconis*: 10+ larvae, all stages: USA: Arizona: Pima Co.: Arizona-Sonora Desert Museum, V-9-1987 (J.G. Rozen, S.L Buchmann); 10+ mature larvae: same except: Catalina State Park, V.-8-1990 (S.L. Buchmann). *Diadasia vallicola*: 10+ late stage larvae: USA: California: Riverside Co.: 18 mi W of Bythe, V-2-1991 (J.G. Rozen). *Diadasina*sp. 2 postdefecating larvae: Argentina: Chaco Prov.: Capitan Solari, I-31-2006 (J. Straka). *Melitoma grisella*: 10+ various larval instars: USA: Nebraska: Keith Co.: Cedar Point Biological Station, VII-20-1988 (J.G. Rozen). *Melitoma marginella*:1 postdefecating larva**:** Mexico: Jalisco: Chemela, XI-7-1986 (J.G. Rozen). *Melitoma segmentaria*:5 mature larvae: Trinidad: Nariva Swamp, X-12-1965 (F.D. Bennett). *Ptilothrix bombiformis*: 2 cast larval skins: USA: Maryland: Prince George Co.: Greenbelt, IX-21, 22-1986 (B. Norden). *Ptilothrix near sumichrasti*: 3 mature lavae: USA: Arizona: Cochise Co.: 8 mi NE of Portal, VIII-18–24- 990 (J.G. Rozen, J. Krieger). *Ptilothrix tricolor*:2 postdefecating larvae: Argentina: Tucumán Prov.: 11 km NW of Cadillal,XII-4-1993 (J.G. Rozen). *Toromelissa nemaglossa*:10+ larvae of all stages: Chile: Atacama Region(III): Huasco Prov. 37 km W of Domeyko, XI11-11-2000 (J.G. Rozen).

*Remarks:* In *Diadasia enavata* (and perhaps in some other species in that genus) the ventral apical mandibular tooth appears missing (Michener 1953: figs 209, 210). Examination of an early stage last larval instar (Fig. 23) shows that it clearly present, but in postdefecating forms it is worn away leaving the mandible with a broad, obliquely truncate apex, bearing a large, adorally directed apical concavity.

## Conclusions and discussion

Because all taxa whose immatures were examined in this study possessed most if not all features of abdominal segments 9 and 10 described above, this character complex strongly supports the relationship of *Ancyloscelis* with the Emphorina, despite the very different mandibles of the two groups*.*

Except for mandibular morphology, there is a strong similarity among not only larval Ancyloscelidina, as represented by *Ancyloscelis apiformis*, and larvae of Emphorina, but also larvae of Exomalopsini (Rozen, in press) and Tetrapediini (Rozen et al. 2006). These similarities include: body shape (protruding venter on abdominal segment 9, dorsally positioned anus, and paired low or virtually absent dorsal body tubercles); spiracle morphology; and head features (excluding mandible morphology).

## References

[B1] Engel MS (2005) Family-group names for bees (Hymenoptera: Apoidea). American Museum of Novitates 3476: 1–33. http://hdl.handle.net/2246/2786 10.1206/0003-0082(2005)476[0001:FNFBHA]2.0.CO;2

[B2] Gonzalez VH Ospina M Palacios E Trujillo E (2007) Nesting habits and rates of parasitism in some bee species of the genera *Ancyloscelis*, *Centris*, and *Euglossa* (Hymenoptera: Apidae) from Colombia. Boletín del Museo de Entomología de la Universidad del Valle 8: 23–29. http://entomologia.univalle.edu.co/boletin/4Gonzalez-etal.pdf

[B3] McGinley RJ (1989) A catalog and review of immature Apoidea (Hymenoptera). Smithsonian Contributions to Zoology494: 1–24. http://www.sil.si.edu/smithsoniancontributions/Zoology/pdf_hi/SCTZ-0494.pdf 10.5479/si.00810282.494

[B4] Michener CD (1953) Comparative morphology and systematic studies of bee larvae with a key to the families of hymenopterous larvae. The University of Kansas Science Bulletin 35: 987-1102.

[B5] Michener CD (1974) Further notes on nests of *Ancyloscelis* (Hymenoptera: Anthophoridae). Journal of the Kansas Entomological Society 41: 19-22.

[B6] Michener CD (2007) Bees of the World, Second Edition*.* Johns Hopkins University Press, Baltimore, MD, 953 pp.

[B7] Roig-Alsina A (1993) Studies of the phylogeny and classification of long-tongued bees (Hymenoptera: Apoidea). The University of Kansas Science Bulletin 55: 124-173.

[B8] Rozen JG Jr (1984) Comparative nesting biology of the bee tribe Exomalopsini (Apoidea: Anthophoridae). American Museum Novitates 2798: 1–37. http://hdl.handle.net/2246/5269

[B9] Rozen JG Jr(in press) Immatures of exomalopsine bees with notes on nesting biology and a tribal key to mature larvae of non-corbiculate, non-parasitic Apinae (Hymenoptera: Apidae). American Museum Novitates.

[B10] Rozen JG Jr Hall HG (2011) Nesting and developmental biology of the cleptoparasitic bee *Stelis ater* (Anthidiini) and its host, *Osmia chalybea* (Osmiini) (Hymenoptera: Megachilidae). American Museum Novitates 3707: 1–38. http://hdl.handle.net/2246/6101 10.1206/3707.2

[B11] Rozen JG Jr Melo GAR Aguiar AJC Alves-dos-Santos I (2006) Nesting biologies and immature stages of the tapinotaspidine bee genera *Monoeca* and *Lanthanomelissa* and of their osirine cleptoparasites *Protosiris* and *Parepeolus* (Hymenoptera: Apidae). Appendix: Taxonomic notes on *Monoeca* and description of a new species of *Protosiri*s, by Melo GAR. American Museum Novitates 3501: 1–60. http://hdl.handle.net/2246/3501 10.1206/0003-0082(2006)501[0001:NBAISO]2.0.CO;2

[B12] Torchio PF (1974) Notes on the biology the biology of *Ancyloscelis armata* Smith and comparisons with other anthophorine bees (Hymenoptera: Anthophoridae). Journal of the Kansas Entomological Society 47: 54-63.

